# Prevailing clone (ST69) of *Vibrio cholerae* O139 in India over 10 years

**DOI:** 10.1186/s13099-017-0210-0

**Published:** 2017-11-06

**Authors:** Shalini Anandan, Naveen Kumar Devanga Ragupathi, Dhiviya Prabaa Muthuirulandi Sethuvel, Suji Thangamani, Balaji Veeraraghavan

**Affiliations:** Department of Clinical Microbiology, Christian Medical College, Vellore, 632004 India

**Keywords:** *Vibrio cholerae* O139, ST69, MLST, Antimicrobial resistance

## Abstract

*Vibrio cholerae* is responsible for the cause of severe life-threatening infection known as cholera. The study aimed to analyze the genetic make-up of *V. cholerae* O139 isolates from India and compare its phylogeny with the global strains. The genome data revealed that all isolates were of same sequence type (ST69) which belongs to seventh pandemic clone, with same virulence gene profile and, antimicrobial resistance gene profile except for two isolates. No known CRISPR repeats were identified in any of these isolates. Three different phages were identified among the isolates. All the isolates were found to harbour *int*_SXT_ and seventh pandemic-specific gene (VC2346). Results from this study enhance our understanding on the persistence of ST69 *V. cholerae* O139 over 20 years.

## Introduction

Historically, only the O1 serogroup of *V. cholerae* has been the causative agent of epidemic cholera. In late 1992, in India and Bangladesh, a novel serogroup designated *V. cholerae* O139 emerged, replacing *V. cholerae* O1, and gave rise to major cholera outbreaks [1]. Based on the whole genome phylogenies of *V. cholerae*, the relation of isolates responsible for global cholera could be understood [2]. Nearly 141 draft genomes of *V. cholerae* O139 are accessible in the NCBI genome projects. Evolution and adaptation mechanism of this pathogen can be demonstrated based on genomic diversity of clinical isolates from various geographical regions and timescale [3]. The current study aims to perform and report the whole genome shotgun sequences of *V. cholerae* O139 isolates for the first time in India. The genomes reported here will help to better understand the evolution and endemicity of cholera in India.

## Methods

### Isolates selection

Clinical strains of *V. cholerae* O139 obtained from the past 20 years (FC1105-2005, FC1225-2001, FC1341-2002, FC1384-2000, FC1817-1994, FC1877-1995, FC2271-1997, FC2273-1998, FC3611a-1999 and FC3611b-1997) were revived from the repository at the Department of Clinical Microbiology, Christian Medical College, Vellore, India. Isolates were identified with standard biochemical methods and were confirmed by agglutination with O139 antisera raised in-house. The genomic DNA was extracted using Qiagen automated DNA extraction method with QIAsymphony DSP Virus/Pathogen Mini Kit (QIAsymphony, Qiagen, Germany). All 10 *V. cholerae* O139 was confirmed based on the 16S rRNA gene, by amplification, sequencing, and BLAST against the NCBI database.

### Next generation sequencing

The whole genome shotgun sequencing for the *V. cholerae* O139 isolates was performed using Ion Torrent (PGM, Life Technologies) with 400-bp read chemistry (Life Technologies, Carlsbad, CA). Genomic DNA libraries were prepared using Ion Plus Fragment Library Kit; (Life Technologies) according to manufacturer’s instructions. AMPure beads were used to purify the genomic libraries and their concentrations were determined using the Qubit 3.0 fluorimeter (Invitrogen, Merelbeke, Belgium). Emulsion PCR was performed on pooled libraries (Ion One Touch Hi-Q 400 Template Kit v2 DL Kit; Life Technologies), and template-positive Ion Sphere particles were enriched using Dynabeads Myone streptavidin C1 beads. Finally, pooled samples were loaded on Ion 318V2 chip for sequencing.

### Genome assembly and annotation

The generated whole genome data were assembled de novo using Assembler SPAdes v.5.0.0.0 embedded in Torrent suite server v.5.0.5. The genome sequence was annotated using PATRIC (Pathosystems Resource Integration Center), the bacterial bioinformatics database and analysis resource (http://www.patricbrc.org) [4], and the NCBI Prokaryotic Genome Automatic Annotation Pipeline (PGAAP) (http://www.ncbi.nlm.nih.gov/genomes/static/Pipeline.html).

### Downstream genome analysis

The whole genome data were analyzed using open access tools at Center for Genomic Epidemiology (CGE) web-based server. Sequence types for the study isolates were determined using MLST 1.8 (https://cge.cbs.dtu.dk//services/MLST/), and antimicrobial resistance were identified using ResFinder 2.1 (https://cge.cbs.dtu.dk//services/ResFinder/) with the 90% threshold for identity and with 60% of minimum length coverage. Genome data was screened for virulence genes through PATRIC database. The presence of plasmids was analyzed using PlasmidFinder 1.3 (https://cge.cbs.dtu.dk//services/PlasmidFinder/) with 95% threshold for identity. The study isolates were further screened for the presence of prophage sequences within the genome using PHAST (PHAge Search Tool) (http://phast.wishartlab.com) [5].

The web-based MyDbFinder 1.0 (https://cge.cbs.dtu.dk/services/MyDbFinder/) was used, in silico to determine the SXT elements (*int*_SXT_), specific integrase genes of class 1 integron (*int*I gene) and specific gene (VC2346) of the seventh pandemic strains with a selected threshold equal to 98% identity as previously described [6].

### Phylogenetic analyses

The phylogenetic analysis was performed based on MLST housekeeping genes (*adk*, *gyr*B, *mdh*, *met*E, *pnt*A, *pur*M, *pyr*C) using the MEGA7 software [7]. The evolutionary history was inferred using the Neighbor-Joining method. The evolutionary distances were computed using the Maximum Composite Likelihood method. goEBURST analysis for the *V. cholerae* O139 strains was performed using PHYLOViZ 1.1 software [8] to identify the relation between study isolates and the global strains with sequence types of *V. cholerae* (ST168-KC895170.1, ST169-KC895206.1, ST170-KJ657169.1, ST182-KC895159.1, ST429-KC895162.1, N16961-AE003852.1, M2140_ST70-CP013315.1, M802_ST71-LT907989.1, M1616_ST73-KC895170.1, M796_ST75-DQ316973.1).

## Results and discussion

### Genome features, virulence and antimicrobial resistance determinants

**Fig. 1:**
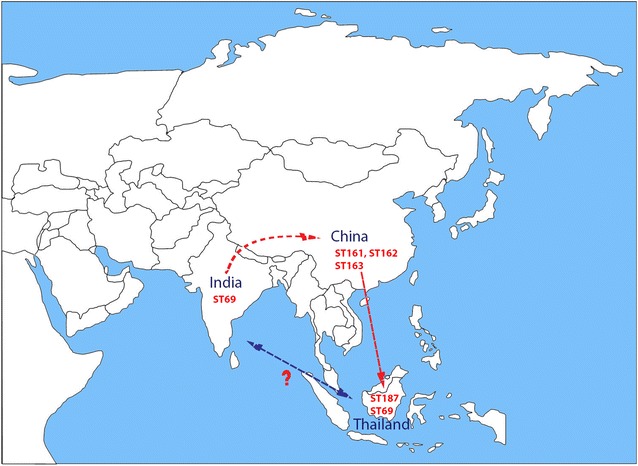
Transmission events inferred for *V. cholerae* O139 sequence types among South Asia

Assembly of the raw reads of the isolates ranged from 69 to 196X coverage (Table 1). NGS revealed that all isolates belong to same sequence type ST69. Similarly in a study by Siriphap et al. [6], most of the seventh pandemic isolates belonged to ST69 and all the clinical strains of O1 and O139 were highly conserved to ST69. Literature study reveals that the Bay of Bengal as a major hub connecting the spread of cholera around the globe and has continually being evolved in South Asia, later spread around the globe in at least three independent but overlapping waves. Besides *V. cholerae* serogroup O1 is widespread throughout the globe, whereas serogroup O139 is almost solely confined to Asia [9].The probable transmission of *V. cholerae* O139 sequence types (ST69, ST187, ST161, ST162 and ST163) that reported elsewhere from South Asia was depicted in Fig. 1.

**Table 1:** Genome characteristics of *Vibrio cholerae* O139 Indian isolates

S. no	Isolate	Year of isolation	GC content (%)	Coverage (X)	CDS	rRNA	tRNA	ARDB	CARD	Sequence type	Confirmed CRISPR	IS elements	Antimicrobial resistance genes	Plasmids	Accession nos.
1	FC1105	2005	47.5	84.7	3785	12	71	6	17	ST69	−	IS3-ISVch4 IS200-IS1004 ISAs1-IS1358 IS481 Tn3-ISShfr9 IS66-ISPsy43 IS5-ISVch8 IS5-ISVch5 IS3-ISVch4 Tn3-ISPsy42	strA, strB, floR, catB9, sul2	−	NGQZ00000000
2	FC1225	2001	47.5	81.7	3796	13	71	2	14	ST69	−	IS3-ISVch4 IS481-ISVch1 Tn3-ISShfr9 IS200-IS1004 ISAs1-IS1358 IS5-ISVch5	catB9	−	NGQY00000000
3	FC1341	2002	47.5	74.7	3763	12	72	2	14	ST69	−	IS3-ISVch4 IS481-ISVch1 Tn3-ISShfr9 IS200-IS1004 ISAs1-IS1358 IS5-ISVch5	catB9	−	NGQX00000000
4	FC1384	2000	47.5	96	3797	12	77	7	18	ST69	−	IS3-ISVch4 IS481-ISVch1 Tn3-ISShfr9 IS200-IS1004 ISAs1-IS1358 IS5-ISVch5	strA, strB, floR, catB9, sul2	−	NGQW00000000
5	FC1817	1994	47.5	69.1	3845	11	75	6	17	ST69	−	IS3-ISVch4 IS481-ISVch1 Tn3-ISShfr9 IS200-IS1004 ISAs1-IS1358 IS5-ISVch5	strA, strB, floR, catB9, sul2	−	NGQV00000000
6	FC1877	1995	47.5	82.4	3750	12	73	6	17	ST69	−	IS3-ISVch4 IS481-ISVch1 Tn3-ISShfr9 IS200-IS1004 ISAs1-IS1358 IS5-ISVch5	strA, strB, floR, catB9, sul2	−	NGQU00000000
7	FC2271	1997	47.5	80.7	3785	14	75	6	17	ST69	−	IS3-ISVch4 IS481-ISVch1 Tn3-ISShfr9 IS200-IS1004 ISAs1-IS1358 IS5-ISVch5	strA, strB, floR, catB9, sul2	−	NGQT00000000
8	FC2273	1998	47.5	77.4	3797	10	71	6	17	ST69	−	IS3-ISVch4 IS481-ISVch1 Tn3-ISShfr9 IS200-IS1004 ISAs1-IS1358 IS5-ISVch5	strA, strB, floR, catB9, sul2	−	NGQS00000000
9	FC3611a	1999	47.5	84.8	3801	11	78	6	17	ST69	−	IS3-ISVch4 IS481-ISVch1 Tn3-ISShfr9 IS200-IS1004 ISAs1-IS1358 IS5-ISVch5	strA, strB, floR, catB9, sul2	−	NGQR00000000
10	FC3611b	1997	47.5	92.9	3819	10	72	6	17	ST69	−	IS3-ISVch4 IS481-ISVch1 Tn3-ISShfr9 IS200-IS1004 ISAs1-IS1358 IS5-ISVch5	strA, strB, floR, catB9, sul2	−	NGQQ00000000

−, negative

ResFinder 2.1 returned same antimicrobial resistance genes for all except two isolates (Table 1). *cat*B9 gene responsible for chloramphenicol resistance was found common in all isolates. *str*A and *str*B (streptomycin resistance), *flo*R (florfenicol/chloramphenicol resistance), and *sul*2 (sulphonamide resistance) genes were identified in all except FC1225 and FC1341 strains. The occurrence of these resistance genes were previously been reported in *V. cholerae* O139 serogroup. Number of antimicrobial resistance genes identified in these genomes as per comprehensive antibiotic resistance database, CARD and antibiotic resistance genes database, ARDB database matching were ~ 17 and ~ 6 genes. PlasmidFinder 1.3 resulted negative for all 10 isolates. Additionally, all 10 isolates were found to possess virulence genes including *ctx*A, *ctx*B, *rtx*, *hly*A, *zot*, *ace*, *tcp*T, *hig*B, *doc*, *par*E.

### Repeat regions and insertional elements

The clusters of regularly interspaced short palindromic repeats (CRISPR) and spacer sequences in the genome were screened using CRISPR finder (http://crispr.u-psud.fr/Server/) [10] and PATRIC database which resulted in no CRISPR regions in these genomes (Table 1).

However, ISfinder (https://www-is.biotoul.fr/blast.php) [11] resulted various IS elements that were listed in Table 1. Most of the IS elements such as IS3-ISVch4, IS200-IS1004, ISAs1-IS1358 (O139), IS481, Tn3-ISShfr9, IS66-ISPsy43, IS5-ISVch8, IS5-ISVch5, IS3-ISVch4, Tn3-ISPsy42 were previously reported from *V. cholerae* genomes. Of these, ISAs1-IS1358 was reported to be from *V. cholerae* O139. Prophage screening results showed that the isolates FC1105, FC1225, FC1341, FC1877, FC3611a and FC3611b were found to have *V. cholerae* phage CTX (NC_015209). The phage K139 (NC_003313) was detected in FC1817, whereas VFJ (NC_021562) was seen in FC2271 and FC2273 respectively.

All study isolates lacked the *int*I gene but were possessed the SXT element (*int*_SXT_) with 99.9% identity to the reference sequence, which was mostly known to be, associated with the sulfamethoxazole and trimethoprim resistant strains [6]. All isolates harboured the seventh pandemic-specific gene (VC2346), suggesting that they belong to the same clonal linage and might originate from a common ancestor of the seventh pandemic strain.

### Phylogenetic analyses

**Fig. 2:**
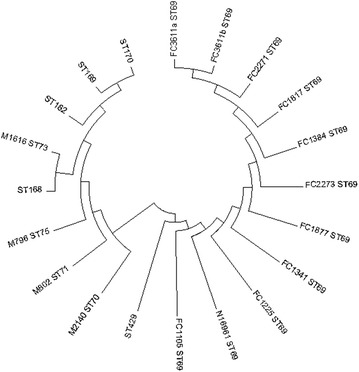
The evolutionary history was inferred using the Neighbor-Joining method. The optimal tree with the sum of branch length = 0.03301416 is shown. The tree is drawn to scale, with branch lengths in the same units as those of the evolutionary distances used to infer the phylogenetic tree. The evolutionary distances were computed using the Maximum Composite Likelihood method and are in the units of the number of base substitutions per site. The analysis involved 20 nucleotide sequences. Codon positions included were 1st + 2nd + 3rd + Noncoding. All positions containing gaps and missing data were eliminated. There were a total of 3215 positions in the final dataset. Evolutionary analyses were conducted in MEGA7

**Fig. 3:**
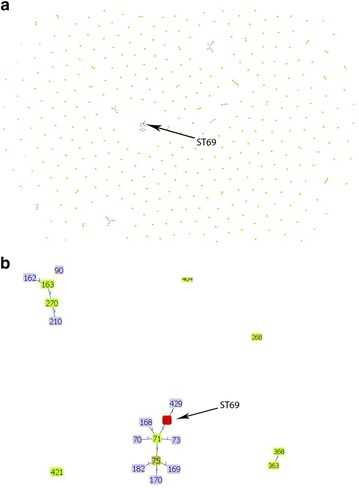
Figure depicting the comparison of *V. cholerae* O139 strains with **a** relation of ST69 with the clones at global level **b** ST69 belongs to one of the few clonal complexes available for *V. cholerae* rather than a singleton

The phylogenetic analysis based on MLST housekeeping genes revealed the relation between ST69 Indian strains and global strains (Fig. 2). All the study isolates are closely related to ST429 originated from M802 (ST71) global isolates. Also, M2140 (ST70), M796 (ST75), ST168, M1616 (ST73), ST169, ST170 and ST182 belong to the same clonal complex with ST71 as the founder type. goEBURST analysis of *V. cholerae* confirmed that ST69 belong to the clonal complex CC0 with the above-mentioned sequence types (Fig. 3).

### Accession numbers

The genome sequences of *V. cholerae* O139 strains have been deposited at DDBJ/ENA/Genbank under the accession numbers NGQQ00000000–NGQZ00000000. The version described in this manuscript is version one.

## Conclusion

To the best of our knowledge, this is the first report of ST69 *V. cholerae* O139 in India. Though there are few reports of clinical *V. cholerae* isolates from India, our study adds new dimension to the earlier finding to show that the sequence type of *V. cholerae* O139 continues to persist in India for over 20 years is of ST69. This implies that clonal expansion of *V. cholerae* O1 seventh pandemic has resulted in ST69 strains. The isolates exhibited same virulence profile but different antimicrobial resistance gene profile. This study helps in understanding the epidemiology of *V. cholerae* and genomic relationship between these isolates with the strains from China and Thailand. Also a continuous surveillance of *V. cholerae* O139 serogroup can provide more insights into the evolution of *V. cholerae*.
